# Abstract of Current Medical Literature

**Published:** 1862-01

**Authors:** 


### EDITORIAL DEPARTMENT.

### ABSTRACT OF CURRENT MEDICAL LITERATURE.

Aortic Aneurism, in which a Communication with the Pulmonary Artery was recognized by means of Physical Diagnosis.—James S——, aged thirty-five, married, a railway porter, was admitted into Queen's Hospital, Birmingham, on May 5th, 1861. For four years he had suffered from piles, and for six months had lost much blood from them, and to this he attributed the debility and wasting for which he sought assistance. Four weeks before admission he had to make a sudden and violent exertion, after which he felt faint for a little while, but thought no more of it. He never had any palpitation or cardiac difficulty; was affected with a little dyspnœa and slight cough with watery expectoration.

*Physical Examination.*—Cardiac dullness increased vertically. Apex seen and felt in the sixth intercostal space. Over the cartilage of the left fourth rib a loud murmur replaced both sounds, that with the second being of a hissing character, and so prolonged as to continue till the commencement of the next first sound, Uusual second sound inaudible there.—Marked thrill at this spot coincident with second murmur. First murmur, a loud bruit de soufflet. Both murmurs heard in the carotid and over the upper chest. At the apex, a single murmur with first sound; normal second sound very distinct. No venous distension. Thrill in the carotids, pulsation of which was visible. Mucus rales in back of both lungs.—Liver enlarged.

From this combination, Dr. Wade concluded—1st, that blood escaped from either the aorta or pulmonary artery during their systole; 2d, that it was probably from the aorta that the blood escaped; 3d, that it did not regurgitate into ether ventricle; 4th, that it regurgitated into one of the auricles or else into the pulmonary artery; 5th, that it did not regurgitate into the left auricle; 6th, that the opening was into the pulmonary artery, rather than into the right auricle; 7th, that the communication was due to aneurismal perforation of the aorta at or near its origin.

The patient stayed in the hospital for two or three weeks, and went back to work, declaring himself well. On the 14th of June he was seized with faintness and violent cardiac perturbation, which continued till the 28th, when he died. The post-mortem examination showed an aneurism of the size of a small hen's egg, very near the root of the aorta, with a rounded, smooth, thickened opening into the pulmonary artery at its origin, and another, fissured, ragged, evidently recent one into the right ventricle. The valves were all healthy. Dr. Wade did not see the patient alive after leaving the hospital.—*London Lancet.*

